# A Rare Case of Recurrent Cutaneous Non-Hodgkin’s Lymphoma in the Extremity: Long-Term Follow-Up and Review of the Literature Written With the Assistance of ChatGPT

**DOI:** 10.7759/cureus.37980

**Published:** 2023-04-22

**Authors:** Taylor Dejong, Jeewanjit Gill, Selay Lam, Sarah Freiburger, Michael Lock

**Affiliations:** 1 Health Sciences, Western University, London, CAN; 2 Family Medicine, University of Ottawa, Ottawa, CAN; 3 Oncology, Western University, London, CAN

**Keywords:** extremity lymphoma, radiation, extranodal lymphoma, small lymphocytic lymphoma, chronic lymphocytic leukemia, chatgpt

## Abstract

Cutaneous involvement of chronic lymphocytic leukemia/small lymphocytic lymphoma (CLL/SLL) is uncommon. We report on a 71-year-old male with a history of CLL of the skin in the distal extremities. The patient presented with eruptions of new lesions on the toes of his feet bilaterally, causing significant pain that limited his mobility. Cutaneous involvement of CLL is a rare presentation, and management recommendations are largely based on case reports with limited follow-up. Furthermore, assessing the duration of response, response rates, and correct sequencing of treatment is difficult due to variable use and doses of treatment. The case was treated in 2001 when newer systemic treatments were not available. Therefore, the results can also be directly related to local treatments. Based on a literature review and this case, this report provides insight into the benefits and risks of local treatment for cutaneous involvement of CLL in the extremities and how radiation can be sequenced with other options such as surgical excision and chemotherapy.

## Introduction

Non-Hodgkin’s lymphoma (NHL) is the most prevalent hematopoietic neoplasm worldwide [1]. It is observed more frequently in individuals aged over 75 years [2]. Genetic factors, including a family history of NHL, have been established as a cause of NHL, as well as certain environmental exposures and lifestyle factors, including, but not limited to, diet and medications [3].

There are different subtypes of NHL, each with different epidemiologies, etiologies, immunophenotypic, genetic, clinical features, and response to therapy [4]. Chronic lymphocytic leukemia/small lymphocytic lymphoma (CLL/SLL) represents approximately 20% of all NHL cases [5]. To ensure consistency in terminology throughout this article, the term CLL will be used to encompass both CLL and SLL. CLL is an indolent malignancy characterized by increased production of mature B-lymphocytes in bone marrow, blood cells, and lymph nodes [6]. The majority of patients are asymptomatic at diagnosis, 10% of patients present with B symptoms, most patients have enlarged and palpable lymphadenopathy, and 20% to 50% of patients present with hepatosplenomegaly on examination [7]. The diagnosis of cutaneous non-Hodgkin’s lymphoma (CNHL) of the extremities is based on clinical presentation, histopathological examination, immunophenotyping, and molecular studies. The most common subtype of CNHL of the extremities is primary cutaneous marginal zone B-cell lymphoma (PCMZL), which typically presents as a slow-growing, painless, and red-to-purple plaque or nodule on the skin.

Treatment for CNHL of the extremities typically involves a combination of surgical excision, radiation therapy, and chemotherapy. The prognosis for this type of cancer varies depending on the stage of the disease and the individual patient's response to treatment. However, overall survival rates are generally favorable.

Regarding cutaneous involvement of CLL, medical literature and standard approach to treatment is lacking, owing to the rarity of this disease presentation. We present a rare case of cutaneous CLL of the extremity, to highlight the details of treatment, toxicity, and natural history.

## Case presentation

A 71-year-old male with a history of CLL and bone marrow involvement presented with new skin eruptions on his toes bilaterally in 2005. His past medical history included chronic obstructive pulmonary disease, gastroesophageal reflux disease, type 2 diabetes mellitus, chronic postherpetic neuralgia, anxiety, benign prostatic hypertrophy, and restless leg syndrome, and his past surgeries included cholecystectomy, Nissen fundoplication, bilateral rotator cuff repair, and orthopedic amputation of his right fifth finger. Until his disease progression in 2005, he was high functioning and was able to work his farm.

In his initial diagnosis in 1999, a biopsy confirmed lymphoid infiltrate with B-cell markers positive for CD-20, CD-79, and CD-5 with monoclonal rearrangement of the immunoglobulin heavy chain gene and no translocation of the *BCL-2* gene. Cryoglobulin was not significantly different from the expected normal values. Bone marrow biopsy confirmed involvement. Flow cytometry confirms monoclonal B-cell population expressing CD-19/CD-5, CD-23, and dim Lambda light chains. Flow immunophenotype was similar to the diagnostic flow on peripheral blood. Laboratory and imaging were within normal limits. He was staged as modified Rai Stage I and Binet Stage B. Clinical risk factors included working as a farmer, his age, and race/ethnicity. He underwent chemotherapy with chlorambucil 14 mg PO once monthly over six months in two cycles with an interval of three months. He had a complete resolution. Within six months of completion of chemotherapy, isolated recurrent cutaneous lesions of CLL erupted on the pinna of his ears bilaterally. These were superficial and involved the helix of both ears extending into the ear lobes. He received 9 MeV electron radiation to the ears with bolus to a dose of 25 Gy in 10 fractions completing treatment in December 2000. He achieved a complete response with long-term mild discoloration in the irradiated area. He had two further recurrences, first in 2001, with head and neck progression, and then in 2003, with neck and right foot masses. Both recurrences were treated with chlorambucil and resulted in complete responses. 

**Figure 1:**
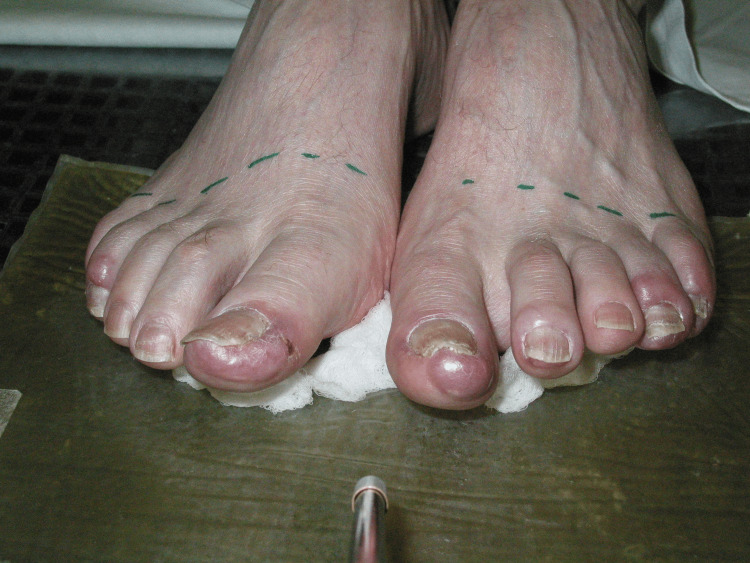
Irregularly demarcated, erythematous, tender nodules, and plaques located on distal phalanges of the feet before treatment.

**Figure 2:**
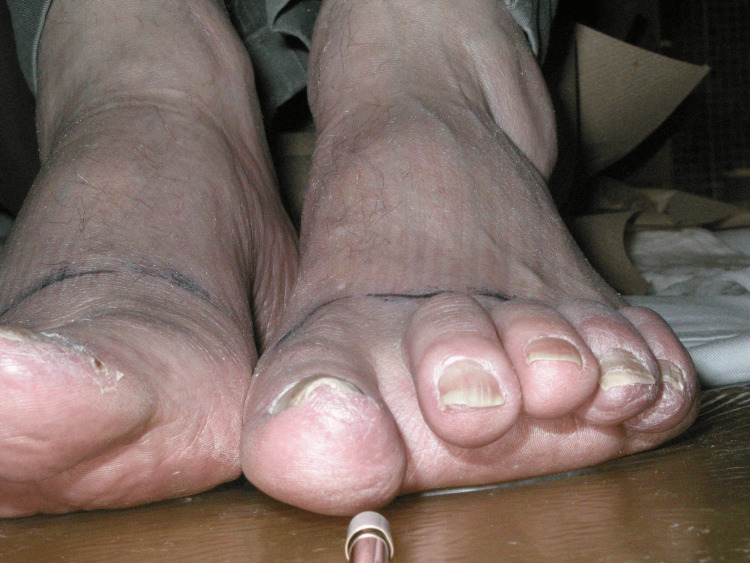
Resolution of cutaneous lesions upon follow-up after treatment.

In January 2005, he was found to have nodules on both feet, with a biopsy confirming lymphoid infiltrate with B-cell markers. The skin lesions were violaceous, erythematous, irregularly demarcated, tender, and thickened firm lesions involving all toes on the left except for the third digit and the right fourth and third toes, without extending beyond the middle phalanges (Figure 1). The recurrence limited the patient’s mobility and ability to perform routine activities. Laboratory and imaging assessments were unremarkable once more including no suggestion of Richter’s transformation. He received 25 Gy in 10 daily fractions of radiation, resulting in complete resolution of gross disease within a month, but also mild tenderness of his toes until three months after treatment. The patient had shingles during the treatment but had no significant lower extremity issues after the three-month assessment, except for the National Cancer Institute Common Terminology Criteria for Adverse Events (NCI CTCAE) grade I erythema and thickening of the skin within the irradiated area (Figure 2). He subsequently had widespread involvement and progressed after fludarabine and then cyclophosphamide, vincristine, and prednisone (CVP). In 2008, the patient was diagnosed with low rectal carcinoma. He underwent a low anterior resection and received leucovorin, 5-fluorouracil, oxaliplatin (FOLFOX), but stopped due to intolerance. In 2009, the patient developed pleural effusion and multiple lung lesions and died in January 2010.

## Discussion

Lymphoma is considered extranodal when the principal expression or the main bulk of the disease is at sites other than lymph nodes and is less common than other presentations [8]. The most common sites for the development of extranodal NHL include the gastrointestinal tract, followed by Waldeyer’s ring, lung, liver, spleen, bone, and skin [9]. As a subgroup of extranodal lymphoma, cutaneous extremity lymphoma is rarely reported in the literature [1]. Although skin infiltration occurs in 3% to 50% of patients with leukemias or lymphomas overall, it is rare in patients with CLL [10]. There are reports to suggest this entity presents in a variety of ways, including erythematous papules, plaques, nodules, and large tumors; however, ulceration is rare [11]. In most observed cases, cutaneous lesions in CLL are an immune dysregulation phenomenon, rather than a direct infiltration by leukemic cells in the skin [10]. There are few reports of cutaneous symptoms being the primary manifestation of CLL and even fewer reports of primary NHL presenting in the foot in the biomedical literature [1,4].

Due to its rare presentation, cutaneous lymphomas may be clinically misdiagnosed for other skin conditions such as squamous cell carcinomas or basal cell carcinomas [12]. When it is unclear, a skin biopsy should be performed to confirm the diagnosis. In our case, there was high clinical suspicion for recurrence of CLL when this case was discussed at multidisciplinary lymphoma rounds based on his clinical presentation and based on a similar previous presentation on the patient’s ears; therefore, a skin biopsy was deemed not warranted. This was the standard practice at the time.

The literature reveals that there are several successful management options for CLL with cutaneous infiltration. The treatment of CNHL of the extremities depends on the stage of disease, the subtype of lymphoma, and the individual patient’s overall health. The treatment may include one or more of the following: (1) localized therapy such as surgery and ultraviolet light B; (2) systemic therapy, including chemotherapy, immunotherapy, or targeted therapy. Options include combination therapies such as electrochemotherapy and bleomycin; (3) watchful waiting; and (4) supportive care for symptoms such as pain, itching, or skin irritation with products such as antihistamines [11,13-15]. However, a localized presentation may allow for local treatment, such as radiation alone to minimize the risk of systemic toxicity; defer the need for systemic options until further, if any, progression; and minimize cost on the healthcare system.

A review of the literature revealed 15 cases of primary cutaneous B-cell lymphoma in the extremities treated with radiotherapy as shown in Table 1. More specifically, a review of the literature revealed four cases of CLL with lesions in the distal extremities treated with radiotherapy as shown in Table 2. Fifteen of the cumulative 19 cases had a response to radiation. At least eight of the cases had extended recurrence-free survivals of over two years with one close to 10 years without recurrence following local treatment. However, follow-up was variable.

**Table 1:** Clinical information of 15 patients with primary cutaneous B-cell lymphoma in the extremities who received radiotherapy as treatment. CHOP, cyclophosphamide, hydroxydaunorubicin, oncovin, and prednisone; M, male; F, female

Reference	Age (years)	Sex	Clinical presentation	Treatment	Response and follow-up
Hembury et al. [16]	62	M	Left arm lesions	Chemotherapy and radiotherapy	No recurrence of lesions at 81-month follow-up
Hembury et al. [16]	62	F	Thigh lesion	Chemotherapy and radiotherapy	Recurrence of symptoms at 16-month follow-up; died of disease 22 months following treatment
Hembury et al. [16]	80	M	Calf lesion	Chemotherapy and radiotherapy	No recurrence of lesion at 26-month follow-up
Hembury et al. [16]	84	M	Leg lesion	Radiotherapy	Recurrence of symptoms at 14-month follow-up
Vasquez-del-Mercado et a.l [17]	74	M	Nodules, with an ulcer, on the right leg and plaques affecting the left leg and both forearms	Radiotherapy localized to right leg followed by CHOP	Good initial response after radiation, but no resolution; the chemotherapy prompted significant improvement
Dasgupta et al. [8]	65	M	Horn-like projections above the right elbow	Six cycles of chemotherapy, in combination with doxorubicin, cyclophosphamide, and vincristine, followed by radiotherapy (50 Gy)	Disease-free at five-year follow-up
Ramanujam et al. [1]	51	F	Lesion on the side of the left foot	Lesion was surgically excised; radiotherapy began six weeks after surgery	No recurrence of lesion at three-month follow-up
Torres-Paoli and Sánchez [18]	87	F	Nodules on the left leg	CHOP followed by radiotherapy	No recurrence of nodules at six-month follow-up
Toberer et al. [19]	66	M	Nodular plaque on the left lower leg	Radiotherapy	The patient spontaneously regressed before receiving radiotherapy, but radiation was still initiated to achieve full clinical remission
Zhao et al. [20]	58	M	Left lower limb plaque	Immunosuppression reduction, CHOP, and local radiotherapy (5,000 cGy)	No signs of relapse at five-month follow-up
Hoefnagel et al. [21]	65	F	Tumor and plaques on the lower part of the left leg	Radiotherapy	No sign of disease at 109-month follow-up
Hoefnagel et al. [21]	35	M	Plaques on the back, upper part of arms, and upper part of the right leg	Radiotherapy	No recurrence of plaques at 76-month follow-up
Hoefnagel et al. [21]	38	F	Plaques on arms, bilaterally	Radiotherapy	No recurrence of disease at 115-month follow-up
Hoefnagel et al. [21]	41	M	Solitary tumor on the upper part of the left leg	Radiotherapy	No signs of disease at 90-month follow-up
Hoefnagel et al. [21]	42	M	Solitary tumor on the left upper arm area	Radiotherapy	No recurrence of tumor at 32-month follow-up

**Table 2:** Clinical information of four patients with cutaneous infiltration by CLL/SLL in the distal extremities who received radiotherapy as treatment. CLL, chronic lymphocytic leukemia; SLL, small lymphocytic lymphoma; M, male; F, female

Reference	Diagnosis	Age (years)	Sex	Clinical presentation	Treatment	Response and follow-up
Morris et al. [22]	SLL	86	M	Swelling of the periungual area of fingers and toes	Radiotherapy followed by six cycles of chlorambucil and rituximab	No significant response to radiation, but the swelling and pain was reduced after the cycles of chlorambucil and rituximab
Freiman et al. [23]	CLL	80	F	Firm symmetric nodules on the distal finger pads	Local electron-beam radiotherapy	Unknown
Caravaglio et al. [24]	CLL	74	M	Bilateral blueish discoloration of toes (blue toe syndrome); ulcerative exophytic plaque on the right second toe	Focal radiation therapy	Toes appeared to be healing well at 18-day follow-up, with no signs of relapse
Simon et al. [25]	CLL	79	M	Subungual tumors involving several fingers and the left big toe	Radiotherapy	75% decrease in size of tumors along with a significant decrease in pain; did not have a further disability in fingers

This case demonstrates the importance of assessing the presence of multifocal skin lesions and extracutaneous disease, as the prognosis and therapeutic approach may differ greatly if either is present. The natural history of this case highlights the need for intermittent treatment for recurrent progressive episodes, and alternative treatments based on toxicity profile should be considered. Patients should be informed that local treatment may not prevent lesions at other locations, and the durability of response to radiation can be short. While the treatment had no significant long-term complications and was well-tolerated, concerns about vascular compromise in the extremities are present. However, these concerns were not an issue in this case nor the literature, and radiation did not compromise or limit future treatment options. The use of higher doses beyond those usually given in this type of lymphoma may improve the durability of the response. Therefore, the selection of treatments to minimize long-term toxicity is a primary consideration, given the often long lifespan of patients.

Despite the isolated local disease, alternative systemic treatments are available that have minimal toxicity and are similarly efficacious. Bruton’s tyrosine kinase (BTK) inhibitors have proven effective in treating CLL by blocking the B-cell receptor (BCR) signaling pathway, preventing the proliferation of malignant and normal B cells [26]. A recent case study by Osmola et al. [27] presents a 60-year-old woman with CLL and skin lesions histologically confirmed as CLL infiltrates. The patient was treated with ibrutinib, which led to the resolution of the lesions, proving the efficacy of BTK inhibitors in the treatment of CLL with cutaneous involvement [27]. BTK inhibitors and other targeted therapy drugs, such as BCL2 inhibitors and PI3K inhibitors, should be considered in future studies in the management of patients with CLL and skin involvement.

This patient subsequently passed away of rectal carcinoma. The patient’s hematological disorder likely increased their risk of developing rectal cancer. This is supported by recent findings proving patients with CLL are at an elevated risk of developing second malignancies [28]. Dennis and Alberts determined that CLL may increase the risk of rectal adenocarcinoma, but further research is needed [28]. Overall, the treatment of CNHL of the extremities is often successful, with a high rate of remission and long-term survival. However, individual outcomes can vary, and patients need to work closely with their healthcare team to determine the most appropriate treatment plan for their specific case. 

ChatGPT was used in the development of this paper. This large language model chatbot was queried on the management of NHL of the extremities (Appendix A). This produced an organized review that was rudimentary but correct. We then assessed the value of ChatGPT in providing a summary of cutaneous NHL of the extremities. ChatGPT provided a concise summary that was deemed useful and used in our introduction (Appendix B). Finally, we sent the entire case history to ChatGPT and asked how it would improve on our management. The response from ChatGPT provided basic recommendations such as suggesting a multidisciplinary approach and the option of palliative care alone. The recommendation of newer treatment options did spark helpful discussion among the authors and reviewers (Appendix C).

## Conclusions

We present an uncommon case of recurrent cutaneous NHL requiring several courses of chemotherapy and adjuvant radiotherapy over a decade. In this case with long-term follow-up, recurrence of CLL manifested only as cutaneous lesions with metachronous involvement of the face, neck, pinna of the ears, and both feet. As these kinds of cutaneous presentations of CLL are rare, a high index of suspicion is warranted to make an accurate clinical diagnosis versus more common skin lesions or cancers. The case highlights the need for iterative use of both systemic and local therapy due to its relapsing nature and the expectations regarding the ability to control the disease. Responses, as in this case, were sometimes not durable and new options need to be investigated, including new agents and higher radiation doses for this uncommon situation.

## Figures and Tables

**Figure 3:**
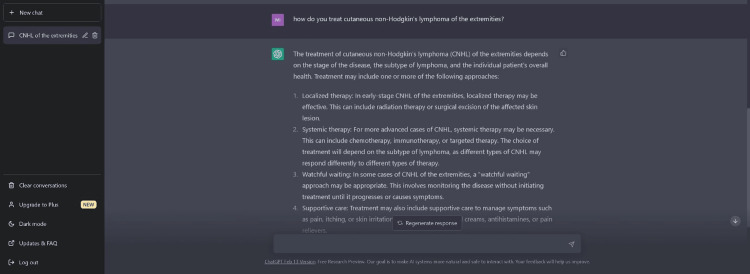
Queried ChatGPT on the management of NHL of the extremities. NHL, non-Hodgkin’s lymphoma

**Figure 4:**
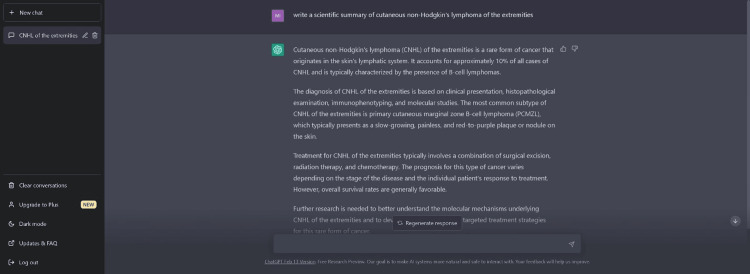
ChatGPT provided a summary of the subject presented in this case, and sections were used verbatim in the final paper.

**Figure 5:**
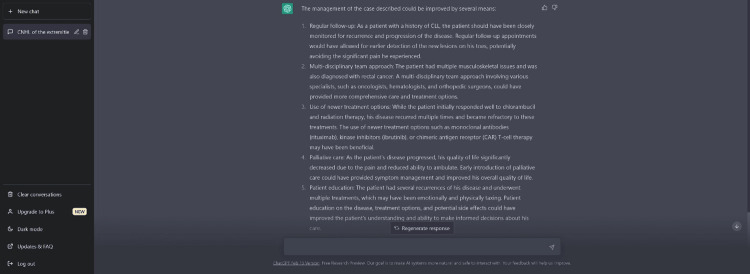
Provided ChatGPT with the full history of the patient and queried how it would improve the management of the case.

## Appendices

Appendix A:  Queried ChatGPT on the management of non-Hodgkin’s lymphoma (NHL) of the extremities

Appendix B: ChatGPT provided a summary of the subject presented in this case, and sections were used verbatim in the final paper

Appendix C: Provided ChatGPT with the full history of the patient and queried how it would improve the management of the case
